# AngioSuite-Assisted Volume Calculation and Coil Use Prediction in the Endovascular Treatment of Tiny Volume Intracranial Aneurysms

**DOI:** 10.1155/2021/5514608

**Published:** 2021-07-29

**Authors:** Zhihua Du, Bin Lv, Xiangyu Cao, Xinfeng Liu, Rongju Zhang, Hui Su, Jun Wang

**Affiliations:** Department of Neurology, Chinese PLA General Hospital, The First Medical Center, Beijing, China

## Abstract

**Methods:**

Thirty-three consecutive patients with 34 TVIAs were prospectively recruited and treated with endovascular techniques. The volume of TVIAs and the required length of coils were calculated by the AngioSuite software before embolization. The treatment efficacy of TVIAs was assessed using the Raymond scale (Rs) and the modified Rankin scale (mRs).

**Results:**

Of the 34 aneurysms with an average volume of 7.16 mm^3^, 13 aneurysms were treated with sole coil embolization, 19 by stent-assisted embolization, and 2 by balloon-assisted embolization. The average coil length was 5.32 cm, and the average packing density was 41.21%. The immediate DSA showed that total occlusion (Rs = 1) was achieved in 15 aneurysms, subtotal (Rs = 2) in 9, and partial (Rs = 3) in 11. Total occlusion was achieved in 30 aneurysms and subtotal in the other 4 aneurysms at 6-month follow-up. Baseline volume and diameter of aneurysms were significantly correlated with the coil length (*r* = 0.801, *P* < 0.001; *r* = 0.711, *P* < 0.001).

**Conclusions:**

Coil embolization of TVIAs was easy to achieve high packing density. According to the data from AngioSuite, relative few coils can increase the safety in procedure and stenting may reduce risk of aneurysmal recurrence.

## 1. Introduction

Intracranial aneurysms (IAs) are commonly acquired cerebral lesions attacking about 2%~3% of the general population, which are associated with significant neurologic impairment and high mortality [1]. Unruptured IAs are more frequent and are being increasingly detected with the advancement of noninvasive imaging techniques with higher resolution [2]. The annual incidence of IA rupture is estimated to be 0.7%, which represents the leading cause of subarachnoid haemorrhage (SAH) [3]. A large cohort study involving 1993 patients with ruptured IAs showed that 83% of the aneurysms were found in anterior circulation locations and the median diameters of these aneurysms were 7 mm [4]. Besides, Grochowski et al. have concluded that rupture of small aneurysms (<5 mm) is a common cause of aneurysmal SAH [5].

Tiny volume intracranial aneurysms (TVIAs) are commonly defined as the aneurysms with size ≤ 3 mm in diameter. Currently, treatment of ruptured TVIAs is still technically challenging and associated with high risk of intraprocedural perforation due to their tiny volumes and thin fragile walls [6]. Endovascular treatments using coiling or stent-assisted coiling techniques emerge as important optional therapies for TVIAs because of their less invasive characteristics [7]. However, excessive coil introduction or insufficient packing will lead to coil compaction and recanalization after IA embolization [8]. The ability to assess the optimal packing density prior to endovascular treatment is fundamentally limited by the lack of accurate calculation of aneurysm volumes [9]. Aneurysm volume measuring is important for treatment planning and packing density calculation [10]. This is especially true for the exact volume calculation of TVIAs due to their extremely limited space for stable coil deployment. Until recently, how TVIA volume should be measured and calculated reaches no consensus [11]. Therefore, based on the guiding role of TVIA volume measuring in endovascular treatment, we propose that the volume of TVIAs is a superior index over diameter in describing the characteristic of a cystic lesion, especially for those aneurysms with irregular appearance. The AngioSuite system is evaluated to be effective in accurate volume calculation of aneurysms based on two- or three-dimensional (2D or 3D) angiograms [9]. However, whether the AngioSuite software has comparable performance in assessing TVIA volume and packing density is currently unknown. Our study is aimed at assessing the ability of the AngioSuite software in calculating TVIA volume and then its role in prospectively selecting suitable coil, predicting packing density and the resultant clinical efficiency of endovascular treatment.

## 2. Materials and Methods

### 2.1. Definition of TVIAs

Tiny intracranial aneurysms are routinely defined as those aneurysms with a maximum diameter equal or less than 3 mm. However, it is not the case for those irregular aneurysm sacs with long and narrow appearances that their maximum diameter is above 3 mm, while the inner space of sacs is extremely small for further endovascular operations. Therefore, we proposed that the volume of TVIA could provide more details and assistance in predicting packing density and choosing suitable coils in real clinical practice. We assumed that the upper limit of TVIA volume could be calculated using the AngioSuite software by separately inputting 3 mm in length, width, and height, and the resultant TVIA volume was equal to 14.14 mm^3^. Therefore, in this study, we defined any aneurysm with volume ≤ 14.14 mm^3^ as TVIA.

### 2.2. Patients and Aneurysms

Thirty-three consecutive patients with 34 TVIAs (volume ≤ 14.14 mm^3^) were prospectively recruited in our center from March 2016 to October2020. Thirty-two patients had ruptured aneurysms and presented acute SAH found by computed tomography (CT) scanning and CT angiography (CTA). They were admitted to our center within 24 h after the onset of SAH. Patient 17 carried an arteriovenous malformation- (AVM-) related unruptured TVIA, and it was embolized when treating the AVM. Patient 31 had 2 TVIAs; the ruptured one was located in the anterior communicating artery (AcoA) and treated at the first administration; the unruptured aneurysm in the middle cerebral artery (MCA) was embolized two weeks later (supplementary table (available here)). The Hunt-Hess grading system (0~V) was applied to evaluate the severity of the clinical symptoms of SAH. The institutional review board and ethics committee of our hospital approved the study protocol and informed consent form. Written informed consent was obtained from all patients.

### 2.3. Angiography and Volume Calculation

Digital subtraction angiography (DSA) and 3D rotational angiography were performed to observe the features of the aneurysms, including the location, diameter, and morphology as well as its anatomic relationship to the parent artery. The aneurysm volume was calculated using the AngioSuite software (Cascade Medical, Knoxville, TN, USA). As previously described, the AngioSuite system is a mathematical algorithm-dependent mobile phone application that uses the loaded biplane angiographic images or tomographic images to automatically calculate the volume of aneurysm only based on one aneurysm diameter [9, 10]. The packing density was also evaluated in this software by calculating the ratio of coil volume to aneurysm volume.

### 2.4. Embolization Procedure

All cases were assessed to be suitable for endovascular coil embolization by the consensus of the neurosurgeons and neurointerventionalists. Surgery was considered as an alternative option to coil embolization only after attempting endovascular treatment. Coiling alone or balloon/stent-assisted coiling treatment strategies were determined based on the parent vessel-aneurysm geometries and dome-to-neck ratio of the TVIAs. We decided to use a stent if the aneurysm was wide necked or had a complex morphology. Stent-assisted coiling was performed as a bailout procedure in case of coil instability or after a failed attempt of simple coiling. For the embolization procedure, all embolization was performed under general anesthesia and the optimal projection was selected to fully expose the aneurysm neck and body and show the relationships to their parent artery and the major artery. A 6-French guiding catheter was delivered via a transfemoral approach with access through the femoral artery using a Siemen's biplane suite (Artis Q) with standard arteriographic and digital roadmap and SmartMask technique. A microcatheter for delivering stents was navigated to the location of aneurysm. Meanwhile, another steam-shaped microcatheter carrying the coil was directed into the aneurysm and partially inserted into the aneurysm cavity. The required length of the coil was calculated before embolization by the AngioSuite software when 30%-40% packing density was simulated. The calculated coil length was referred during coiling to ensure that the used length did not obviously exceed the calculated length in real practice. After one or more coils were released to fill the aneurysmal cavity, stent was partially released to prevent coil protrusion into the parent artery. Stent was completely released after the coil deployment was discontinued and the coil was isolated. Afterwards, the coil pusher was secured and the microcatheter containing the coil was slightly withdrawn. The coil delivery microcatheters used in this study included SL-10 (Stryker) and Echelon 10 (Covidien). The main coils used in this study were Axium coils (Covidien) and Target coils (Stryker Neurovascular, Freemont, CA). The type of stent was chosen depending on the surgeon's preference and the patient's vascular anatomy. Five types of stents were used in this study, including Neuroform EZ, Solitaire AB, Leo-Leo & Leo baby, Atlas, and Lvis Jr stents. Balloon-assisted coiling was performed in two patients to prevent prolapse of the coil loop, and the degree of balloon inflation varied during coil placement to allow for microcatheter movement. Detailed magnified view of angiography was performed at the end of each procedure to determine degree of embolization and to identify possible complications such as rupture or thrombosis.

### 2.5. Anticoagulation and Antiplatelet Management

Heparin was intravenously administrated after placement of sheath at a dose of 0.6~0.8 mg/kg body weight, followed by hourly boluses (half of the dose of the baseline dose, but no less than 10 mg). The activated clotting time was maintained at 2~3 times the baseline throughout the procedure. If stent placement was scheduled for patients with ruptured aneurysm, a loading dose of 300 mg clopidogrel and 300 mg aspirin was administrated 2 hours before stenting; 100 mg aspirin and 75 mg clopidogrel were administered 24 hours after the procedure. For patients that underwent emergent stenting, tirofiban was intravenously injected at a dose of 0.005 mg/kg body weight before releasing the stent and this dose was maintained for 24 hours. Afterwards, tirofiban infusion was progressively decreased and changed to the regimens containing 100 mg aspirin and 75 mg clopidogrel. Aspirin (100 mg daily) and clopidogrel (75 mg daily) were maintained for 1~3 months after the operations based on the selection of stent type, followed by aspirin alone (100 mg daily) afterwards for 3~6months.

### 2.6. Outcome Evaluation and Follow-Up

A conventional follow-up using catheter angiography was performed 6 months after the embolization. The degree of aneurysm embolization was assessed using the Raymond scale (Rs) immediately after the embolization and 6 months postoperatively. Rs was classified as complete occlusion of the sac and neck of the aneurysm (Rs = 1), near-complete occlusion (the sac was occluded but a neck remnant was suspected or obviously present) (Rs = 2), and incomplete occlusion (there was persistent opacification of a sac remnant) (Rs = 3). Clinical outcome at follow-up was assessed using the modified Rankin scale (mRs) graded from 0 to 6.

### 2.7. Data Analysis

All data analysis was performed in SPSS 26.0 software. Demographic and clinical characteristic data relating to continuous variables are expressed as means ± SD. Correlations between volume or diameter of aneurysms and coil length used or packing density were analyzed using Pearson analysis. A *P* value less than 0.05 is considered statistically significant.

## 3. Results and Discussion

### 3.1. Baseline Demographics and TVIA Characteristics

Thirty-four patients, consisting of 21 (61.8%) females and 13 (38.2%) males, were prospectively recruited in this study with an average age of 60.24 ± 12.72 (ranging 32~85) years. These patients presented SAH due to tiny intracranial aneurysms with Hunt-Hess grade I in 18 cases, grade II in 11 cases, and grade III in 3 cases. These aneurysms were predominately located in AcoA (13 cases), MCA (8 cases), and posterior communicating artery (PcoA) (6 cases), accounting for 79.4% of the TVIAs. As calculated by the AngioSuite software, the average diameter and volume of the aneurysms were 2.95 mm (1.76~5.0 mm) and 7.16 mm^3^ (1.6~13.82 mm^3^), respectively. 21 (61.8%) aneurysms had diameter below 3 mm (Table 1).

### 3.2. Treatment Efficacy

For the treatment of intracranial aneurysms, 13 of them were managed with single coil embolization, 19 with stent-assisted embolization, and 2 with balloon-assisted embolization. The average length of coils was 5.12 ± 3.18 (1~12) cm, and packing density was 41.31 ± 16.55 (17~72) %. One case of stent thrombosis occurred during the procedures, and it was replaced with another Atlas stent. An aneurysm ruptured during the operation, and it was embolized with coil. All other aneurysms were treated successfully. Complete occlusion was achieved in 15 patients (46.2%), while 9 (30.8%) and 11 (23.1%) patients achieved near-complete occlusion and incomplete occlusion, respectively (Table 2).

### 3.3. Angiographic Follow-Up

**Figure 1:**
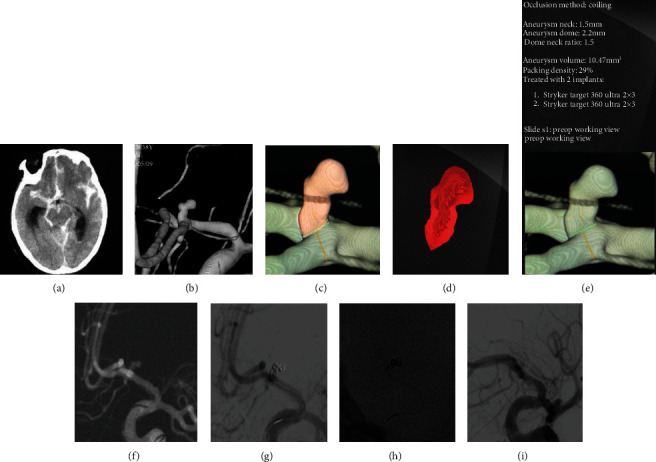
Case presentation of patient 6, female, 58 years old. She presented with (a) SAH on noncontrast head CT and (b) a ruptured irregular AcoA aneurysm on 3D reconstruction image of DSA. The AngioSuite software was used to configure and evaluate the volume of aneurysm before the embolization procedure (c–e). Based on the AngioSuite data, embolization with 2 coils (two targets 2-3 mm) could achieve sufficient immediate occlusion (Raymond 2) (f, g). Sparse coils showed on nonsubtracted image (h). Six-month follow-up showed a complete occlusion (Raymond 1) (i).

All patients were followed up six months postoperatively; complete occlusion was achieved in 30 (88.2%) of 34 aneurysms and near-complete occlusion was achieved in 4 (11.8%) aneurysms. 33 of the aneurysms had decreased Rs grade compared to immediately postoperative outcome, and one aneurysm had no change in Rs grading. At six months of follow-up, 31 (93.9%) of the patients had no neurological complications as mRs scoring 0, and the remaining two patients (aged over 80 years) had mild symptoms but no functional deficiencies with mRs scoring 1 (Table 2). No migration of stents or coils occurred at follow-up compared with immediate position after embolization. None of the aneurysms showed recanalization requiring additional treatment. The case presentation of case 6 (supplementary table (available here)) is shown in Figure 1.

### 3.4. Roles of Aneurysm Volume Measurement in Guiding Coil Embolization

**Figure 2:**
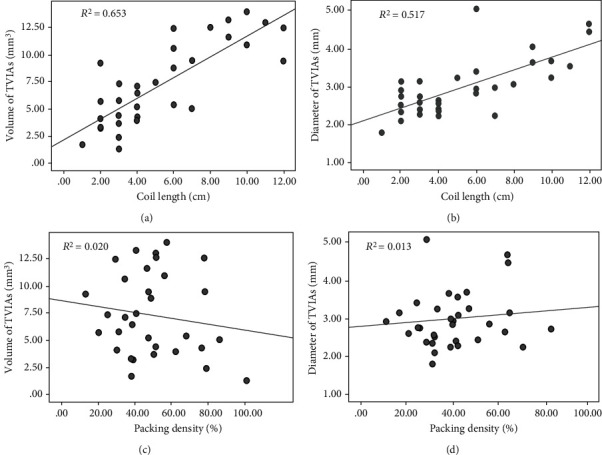
Correlation analyses between aneurysm volume or diameter and length of coils or packing density of coils during embolization. Scatter plots of (a) volume of TVIAs against coil length, (b) diameter of TVIAs *vs.* coil length, (c) volume of TVIAs *vs*. packing density, and (d) diameter of TVIAs *vs*. packing density.

We further explored the correlation between volumes of aneurysms and coil length used during embolization by Pearson analysis. The results showed that baseline volumes (calculated by the AngioSuite software) and diameters of TVIAs were significantly correlated with the coil length used during embolization (*r* = 0.801 and *P* < 0.001 and *r* = 0.711 and *P* < 0.001, respectively), indicating that aneurysmal volume as determined by AngioSuite was a preferable parameter in guiding the coil embolization procedure. In addition, no significant correlations were shown between volume or diameter and packing density of the aneurysms (*r* = −0.163, *P* = 0.363; *r* = 0.099, *P* = 0.585) (Figure 2).

## 4. Discussion

With the wide application of 3D rotational angiography, small and tiny aneurysms have been diagnosed more frequently. Tiny aneurysms have been expected to have a high rate of ruptures up to 15% [12, 13]. Unfortunately, no specific recommendations for treating TVIAs are available up to date [2]. In recent years, coil embolization with the assistance of stent or balloon offers a reliable and safe strategy for the treatment of tiny aneurysms with the advancement of endovascular techniques. However, TVIA embolization has higher risk of procedural complications than other intracranial aneurysms mainly due to excessive coils with limited space to obtain a stable microcatheter position for coil deployment [14]. Meanwhile, an optimal packing density during endovascular treatment is necessary for preventing coil compaction and improving prognosis of these patients. In this study, we used the AngioSuite software to calculate the TVIA volume and the required coil length to ensure sufficient packing density. The AngioSuite-based volume estimation provides efficient assistance for assessing the required coil length and packing density, in turn reducing the rupture during embolization and recurrence of TVIAs in the follow-up.

The volume of intracranial aneurysm is a very important parameter for endovascular treatment planning and embolization course, especially for those irregular cystic lesions. An inaccurate measurement in a millimeter level may cause higher risk of rupture of TVIAs and secondary misuse of expensive endovascular devices [10]. Currently, the accurate calculation of aneurysm volume remains a challenge. Woodward and Forsberg concluded that volume calculation based on 3D images was greatly affected by identification of threshold windows dependent on the operator, while 2D image-based volume calculations can be adversely influenced by lacking reliable markers, irregular aneurysm shapes, and overly simplistic spherical or elliptical geometric models [9]. Therefore, how to size the intracranial aneurysm has been disputed and discussed over the past years. A study by Behme et al. reported that the most exact aneurysm measurement can be achieved on a 2D DSA image with a short objective-to-detector distance adjusted according to a previous 3D run [11]. Meanwhile, the AngioSuite software is proposed to be an accurate method to calculate aneurysm volumes in comparison with AngioCalc or other specialized software based on rotational angiography [9, 10]. The AngioSuite mathematical system runs based on bidimensional angiographic images. In this study, we used the AngioSuite software to calculate TVIAs' volume and the required length of coil that reached sufficient packing density. The coil length has more prominent influence on the packing density for TVIAs than for the large or giant IAs. Currently, most of the coil lengths are measured in centimeters, and an increase of 1 cm in coil length leads to obvious increase in packing density for TVIAs, while it may have no notable effect on an aneurysm with a diameter over 5 mm.

Most authors believe that increased packing densities result in lower compaction and decreased aneurysmal recurrence. Piotin et al. [15] noted a 44.4% recurrence rate in aneurysms with packing densities ≤ 25% and 29.8% in packing densities > 25%. Sluzewski et al. [16] showed that aneurysms packed to >24% did not show compaction at 6 months of follow-up. However, “the more the better” would not be always right in the embolization procedure. Intraprocedural rupture is a fearful complication in the endovascular treatment of TVIAs [17–19]. Nguyen et al. [17] found that endovascular coil embolization of very small (≤3 mm) ruptured cerebral aneurysms is 5 times more likely to result in procedure-related rupture compared with larger aneurysms. The smaller size of the aneurysm sac limits the movement of the microcatheter; thus, any unexpected movement during catheter positioning or coil deployment can result in rupture of the aneurysm sac [14]. Endovascular devices such as coils or stents may be oversized, which confer a higher rupture risk. Tiny volume is liable to reach sufficient packing density even when fewer coils are used. Chen et al. [20] found that aneurysmal size was an independent influencing factor for immediate angiographic outcome after embolization in patients with TVIAs. A study by Maeda et al. [21] suggested that the length of all coils should not be longer than 10 cm in the embolization of TVIAs in terms of safety. Lu et al. [22] believed that complete coil occlusion of the aneurysm sac was not necessary to achieve better long-term outcomes. We currently consider that >30% is a sufficient packing density for the embolization and approximate length of coils should be selected according to the data of AngioSuite. An acceptable immediate occlusion (Rs 1 and Rs 2) rate (70.6%) was obtained by a 5.32 cm average length of coils and 41.31% packing density in our series. We also found that there is a significant linear correlation between the length of coils and the volume of aneurysms.

In this study, we used the AngioSuite software before the procedure aiming to minimize the use of coils and increase the safety during the operations. Meanwhile, stent deployment enhanced the flow diverting and further reduced the use of coil. For case 11 (supplementary table (available here)), only 2 cm coil was used and the rupture risk of the aneurysm during embolization potentially decreased, while the resultant thrombotic complications could be efficiently treated with new antiplatelet drugs. Although fewer coils could reduce the risks of rupture during embolization, tiny volume is still an adverse factor for coil stabilization. Assistant devices such as high elasticity balloon and stent are needed sometimes. The placement of a balloon at the side of the aneurysm neck to stop haemorrhage has been advocated; however, the use of additional adjunctive devices during treatment of very small IAs has been proposed to associate with increased complication rates in some studies [23, 24]. Stent placement is helpful in packing coils and preventing coil escape, even playing a role in flow diverting. Stents across the neck of an aneurysm redirect the blood flow and decrease intra-aneurysmal flow velocity by disturbing the inflow. The reduced aneurysmal flow induces stasis and consequently thrombosis of the aneurysm [25]. Tateshima et al. [26] investigated the alterations in intra-aneurysmal hemodynamics following the placement of high-porosity open-cell stents across the necks of aneurysms and found that the placement of a single Neuroform stent reduced the intra-aneurysmal flow velocity by 22%-64%. In another study, the authors assessed the hemodynamic changes inside aneurysms induced by the implantation of conventional stents and found that the velocity of the jet flow entering the aneurysm and wall shear stresses on the aneurysm were significantly reduced by the deployment of a stent [27]. Even then, stent placement also associated with higher thrombosis complication in the endovascular treatment of TVIAs [28]. In our case series, more than half of the cases (19/34) underwent stent-assisted embolization procedure and one case of in-stent thrombosis was treated immediately. In our experience, intravenous transfusion of tirofiban (0.005 mg/kg/h) was prescribed before stent deployment and continued for 24 hours, and then, stepwise reduced doses were recommended until dual oral antiplatelet regimens (aspirin 100 mg and clopidogrel 75 mg per day) were given at least 3 times. In a study by Wu et al. [13], the patients with ruptured TVIAs were prescribed with dual antiplatelet therapy (aspirin 300 mg and clopidogrel 300 mg) 2 hours prior to the Lvis stent-assisted embolization, which also achieved safe outcomes. However, some cases with high grade of Fisher scale or with intracranial hematoma who had high possibility of surgery would not be indicated for stenting.

## 5. Conclusions

Embolization was reliable to achieve a high packing density and occlusion rate in treating TVIAs. According to the data from AngioSuite, relative few coils can increase the safety in procedure. For those patients with mild TVIAs, stent-assisted coil may be a helpful option to reduce the risk of aneurysm recurrence.

## Figures and Tables

**Table 1:** Baseline characteristics and aneurysmal features.

Clinical characteristics	Values
Gender (M/F)	13/21
Age (years)	60.24 ± 12.72
HH grade	
1	18
2	11
3	3
Unruptured	2
Aneurysmal location	
AcoA	13
PcoA	6
CPco	3
MCA	8
ACA	2
BA	2
Aneurysmal size	
Diameter (mm)	2.95 ± 0.74
Diameter ≤ 3 mm (cases)	21
Volumes (mm^3^)	7.16 ± 3.74 (1.6~13.82)

Note: Emb: coil embolization; MCA: middle cerebral artery; BA: basilar artery; PcoA: posterior communicating artery; AcoA: anterior communicating artery; CPco: communicating segment of internal carotid artery; ACA: anterior cerebral artery.

**Table 2:** Embolization procedure and angiographic follow-up of the TVIAs.

Characteristics	Values
Embolization	
Single coil embolization	13
Stent-assisted	19
Balloon-assisted	2
Stent types	
Leo	7
Neuroform	4
Atlas	4
Solitaire AB	3
Lvis Jr	1
Coil types	
Target	29
Axium	4
Premier	1
Clinical outcomes	
Coil length (cm)	5.32 ± 3.18
Packing density (%)	41.31 ± 16.55
Immediate Rs	
1	15
2	9
3	10
Follow-up Rs	
1	30
2	4
Follow-up mRs	
0	32
1	2

Note: Emb: coil embolization; EZ: Neuroform stent; SAB: Solitaire AB stent; Leo: Leo & Leo baby stent; Ta: Target coil; Ax: Axium coil; Rs: Raymond scale; mRs: modified Rankin scale.

## Data Availability

The datasets used during the present study are available from the corresponding author upon reasonable request.

## Supplementary Materials

Supplementary Materials Supplementary Table: baseline characteristics and procedure/follow-up occlusion outcomes.
