# Supplementary material for: AngioSuite-Assisted Volume Calculation and Coil Use Prediction in the Endovascular Treatment of Tiny Volume Intracranial Aneurysms
Source: Biomed Res Int. 2021 Jul 29;2021:5514608. doi: 10.1155/2021/5514608 (PMC8342139; doi:10.1155/2021/5514608)
Supplement: Supplementary Materials — Supplementary Table: baseline characteristics and procedure/follow-up occlusion outcomes. [file 5514608.f1.docx]

# Biomedical Research International

**AngioSuite-assisted volume calculation and coil use prediction in the endovascular treatment of tiny volume intracranial aneurysms**

**Supplementary Table Baseline characteristics and procedure/follow-up occlusion outcomes**

| Patients | Gender | Age (yesr) | Hunt-Hess grade | Location | Diameter  (mm) | Volume（mm^3^） | Emb type | First coil | Coil length (cm) | Packing density  (%) | Immediate Rs | Follow-up (6 m) Rs | mRS  (6 m) |
| --- | --- | --- | --- | --- | --- | --- | --- | --- | --- | --- | --- | --- | --- |
| 1 | F | 55 | 2 | MCA | 2.6 | 4.17 | Single Emb | Ax2-2 | 4 | 64 | 2 | 1 | 0 |
| 2 | M | 57 | 1 | BA | 2.7 | 5.65 | Single Emb | Ta2-3 | 3 | 26 | 3 | 2 | 0 |
| 3 | F | 61 | 1 | PcoA | 3.2 | 7.32 | Lvis Jr | Ta2-4 | 5 | 34 | 3 | 1 | 0 |
| 4 | F | 66 | 1 | AcoA | 2.8 | 5.28 | Single Emb | Ta2.5-4 | 6 | 57 | 2 | 1 | 0 |
| 5 | F | 85 | 3 | CPco | 4 | 13.09 | Single Emb | Ta3-4 | 9 | 34 | 2 | 2 | 1 |
| 6 | F | 58 | 2 | AcoA | 5 | 10.47 | Single Emb | Ta2-3 | 6 | 29 | 2 | 1 | 0 |
| 7 | M | 55 | 1 | MCA | 2.4 | 3.85 | EZ | Ta1.5-2 | 4 | 52 | 1 | 1 | 0 |
| 8 | F | 51 | 2 | AcoA | 3.2 | 13.82 | SAB | Ta3-4 | 10 | 48 | 1 | 1 | 0 |
| 9 | F | 53 | 2 | CPco | 4.6 | 12.36 | EZ | Ax3-4 | 12 | 65 | 1 | 1 | 0 |
| 10 | F | 81 | 2 | CPco | 3.5 | 12.83 | Hypoform | Ta3-4 | 11 | 43 | 1 | 1 | 0 |
| 11 | M | 48 | 1 | MCA | 3.1 | 5.6 | EZ | Ta1.5-2 | 2 | 17 | 1 | 1 | 0 |
| 12 | M | 87 | 3 | MCA | 2.2 | 4.92 | Leo | Ta2-2 | 7 | 72 | 1 | 1 | 1 |
| 13 | M | 54 | 1 | ACA | 3.6 | 11.47 | Leo | Ta2-2 | 9 | 39 | 3 | 1 | 0 |
| 14 | F | 55 | 1 | MCA | 2.24 | 4.3 | Leo | Ax2-2 | 3 | 43 | 3 | 1 | 0 |
| 15 | F | 57 | 2 | PcoA | 2.71 | 4 | SAB | Ta2-2 | 2 | 25.3 | 3 | 1 | 0 |
| 16 | F | 56 | 2 | AcoA | 2.9 | 8.7 | Single Emb | Ta3-4 | 6 | 40.8 | 2 | 1 | 0 |
| 17 | M | 55 | AVM concomitant | MCA-M2 | 1.76 | 1.6 | Single Emb | Ta1.5-1 | 1 | 31.7 | 2 | 1 | 0 |
| 18 | F | 80 | 2 | PcoA | 3.36 | 12.3 | EZ | Ta3-4 | 6 | 24.7 | 3 | 1 | 0 |
| 19 | M | 66 | 1 | BA | 4.4 | 9.3 | Atlas | Ta2.5-4 | 12 | 65.4 | 1 | 1 | 0 |
| 20 | F | 56 | 1 | PcoA | 2.47 | 3.1 | Single Emb | Ta2-2 | 2 | 32.7 | 3 | 2 | 0 |
| 21 | M | 64 | 1 | AcoA | 2.87 | 9.1 | Atlas | Ta2-2 | 2 | 11.1 | 3 | 1 | 0 |
| 22 | F | 65 | 1 | AcoA | 2.2 | 5.1 | Leo | Ta1.5-3 | 4 | 39.7 | 2 | 1 | 0 |
| 23 | M | 57 | 3 | AcoA | 2.36 | 3.6 | Single Emb | Ta1.5-3 | 3 | 42.2 | 2 | 1 | 0 |
| 24 | F | 63 | 2 | AcoA | 2.52 | 6.3 | Single Emb | Ta2-2 | 4 | 32.2 | 1 | 1 | 0 |
| 25 | F | 56 | 3 | AcoA | 2.94 | 9.3 | SAB | Ta2.5-4 | 7 | 39.9 | 1 | 1 | 0 |
| 26 | F | 32 | 1 | ACA-A1 | 3.1 | 2.3 | Leo | Ta1.5-2 | 3 | 66.1 | 1 | 1 | 0 |
| 27 | F | 62 | 1 | PcoA | 2.56 | 7.2 | Atlas | Ta2-3 | 3 | 21.1 | 3 | 1 | 0 |
| 28 | M | 32 | 1 | AcoA | 3.03 | 12.4 | Leo | Ax3-4 | 8 | 43.2 | 2 | 1 | 0 |
| 29 | M | 63 | 2 | AcoA | 3.64 | 10.8 | Hyperform | Ta2.5-4 | 10 | 46.9 | 1 | 1 | 0 |
| 30 | M | 87 | 2 | MCA | 2.68 | 1.2 | Leo | Ta1.5-2 | 3 | 84.5 | 1 | 1 | 0 |
| 31 | F | 57 | 1 | AcoA | 2.8 | 8.7 | Single Emb | Prime2-4 | 6 | 40.8 | 1 | 1 | 0 |
|  |  |  | Non-offending vessel | MCA-M2 | 2.3 | 3.2 | Atlas | Ta1.5-2 | 2 | 31.7 | 3 | 1 | 0 |
| 32 | F | 53 | 2 | AcoA | 2.06 | 3.1 | Single Emb | Ta1.5-2 | 2 | 32.7 | 1 | 1 | 0 |
| 33 | M | 61 | 1 | PcoA | 2.33 | 7 | Single Emb | Ta2-4 | 4 | 29 | 3 | 2 | 0 |

Emb: coil embolization; R: Raymond scale; MCA: middle cerebral artery; BA: basilar artery; PcoA: posterior communicating artery; AcoA: anterior communicating artery; CPco: communicating segment of internal carotid artery; ACA: anterior cerebral artery; EZ: Neuroform stent; SAB: Solitaire AB stent; Leo: Leo & Leo baby stent; Ta: Target coil; Ax: Axium coil; mRS: modified Rankin Scale; AVM: arteriovenous malformation.
